# Understanding transmission and control of the pork tapeworm with CystiAgent: a spatially explicit agent-based model

**DOI:** 10.1186/s13071-020-04226-8

**Published:** 2020-07-24

**Authors:** Ian W. Pray, Wayne Wakeland, William Pan, William E. Lambert, Hector H. Garcia, Armando E. Gonzalez, Seth E. O’Neal, for the Cysticercosis Working Group in Peru

**Affiliations:** 1 School of Public Health, Oregon Health & Science University and Portland State University, Portland, OR USA; 2 Systems Science Program, Portland State University, Portland, OR USA; 3 Global Health Institute, Duke University, Durham, NC USA; 4 School of Sciences, Department of Microbiology, Universidad Peruana Cayetano Heredia, Lima, Peru; 5 School of Veterinary Medicine, Universidad Nacional Mayor de San Marcos, Lima, Peru

**Keywords:** *Taenia solium*, Cysticercosis, Agent-based models, Infectious disease modeling, Peru

## Abstract

**Background:**

The pork tapeworm, *Taenia solium*, is a serious public health problem in rural low-resource areas of Latin America, Africa and Asia, where the associated conditions of nuerocysticercosis (NCC) and porcine cysticercosis cause substantial health and economic harms. An accurate and validated transmission model for *T. solium* would serve as an important new tool for control and elimination, as it would allow for comparison of available intervention strategies, and prioritization of the most effective strategies for control and elimination efforts.

**Methods:**

We developed a spatially-explicit agent-based model (ABM) for *T. solium* (“CystiAgent”) that differs from prior *T. solium* models by including a spatial framework and behavioral parameters such as pig roaming, open human defecation, and human travel. In this article, we introduce the structure and function of the model, describe the data sources used to parameterize the model, and apply sensitivity analyses (Latin hypercube sampling-partial rank correlation coefficient (LHS-PRCC)) to evaluate model parameters.

**Results:**

LHS-PRCC analysis of CystiAgent found that the parameters with the greatest impact on model uncertainty were the roaming range of pigs, the infectious duration of human taeniasis, use of latrines, and the set of “tuning” parameters defining the probabilities of infection in humans and pigs given exposure to *T. solium.*

**Conclusions:**

CystiAgent is a novel ABM that has the ability to model spatial and behavioral features of *T. solium* transmission not available in other models. There is a small set of impactful model parameters that contribute uncertainty to the model and may impact the accuracy of model projections. Field and laboratory studies to better understand these key components of transmission may help reduce uncertainty, while current applications of CystiAgent may consider calibration of these parameters to improve model performance. These results will ultimately allow for improved interpretation of model validation results, and usage of the model to compare available control and elimination strategies for *T. solium*.
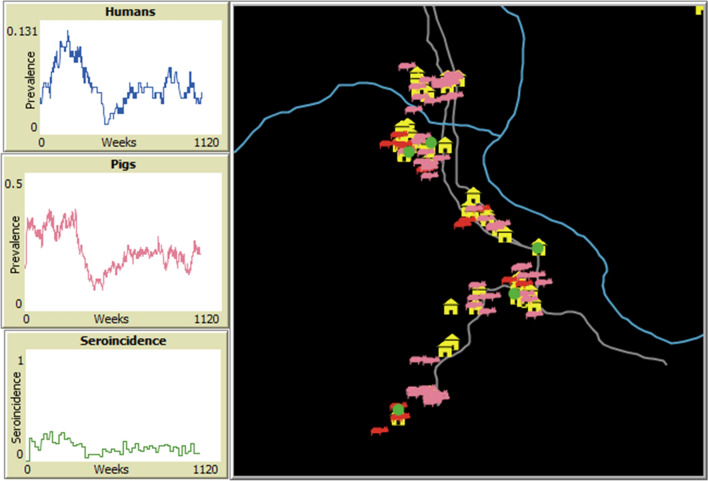

## Background

The pork tapeworm, *Taenia solium*, remains a major public health concern in poor rural areas of the world. In endemic regions, up to one third of seizure disorders are attributed to neurocysticercosis (NCC), a severe neurological infection caused by the parasite [1, 2], and lost income from infected pork lead to financial losses for pig farmers [3]. Humans acquire the adult-stage intestinal tapeworm (human taeniasis) by consuming raw or undercooked pork that is infected with intermediate-stage larval cysts, while pigs acquire this cyst infection (porcine cysticercosis) through contact with eggs present in the feces of infected humans. NCC is a larval infection of the central nervous system that occurs in humans when the eggs are ingested through fecal-oral contact.

Control and elimination of *T. solium* transmission in endemic areas is now known to be achievable [4, 5] through strategic application of available drugs to treat human taeniasis [6, 7] and porcine cysticercosis [8], and a vaccine to prevent infection in pigs [5, 9]. Despite these effective tools, there remains limited evidence on which to base decisions about which interventions or strategic combinations of interventions are most likely to be successful in different endemic regions. Prospective trials that compare available strategies have made important contributions [4], but have been too costly to execute on the scale needed for policy decisions. The World Health Organization (WHO) recently called upon the use of transmission modeling to help address this evidence gap. In 2012, WHO called for *T. solium* models to be deployed to identify a set of validated strategies that could be implemented in several countries by 2020 [10], and recently, the 2030 goals reinforced modeling as a priority for *T. solium* control and elimination [11].

In response to these calls, a variety of *T. solium* models have been developed in recent years [12–16]. These existing models, like many traditional infectious disease models, rely on assumptions of spatial homogeneity, closed populations, and parameter values that are averaged across large populations. Transmission of *T. solium*, however, is uniquely difficult to model under traditional assumptions due to the complex social, biological, and environmental factors that perpetuate transmission in endemic areas. Local variations in pig-raising practices, sanitation, diet, and migration all interact to create locally specific transmission patterns that differ from one endemic village to the next [17]. Even within villages, spatial heterogeneities caused by pig-roaming patterns and open defecation cause clustering that is important for a model to capture [18–20]. Importantly, incorporating underlying spatial and biological processes of *T. solium* transmission was highlighted in a recent report on the WHO 2030 goals [11], and there is evidence that models that fail to account for these heterogeneities are susceptible to overestimating the effect of control interventions [21] and yielding unrealistic predictions for achieving control and elimination targets [22].

To avoid the pitfalls described above, complex ecological systems like *T. solium* transmission are well-suited for agent-based models (ABM). ABMs are increasingly used for modeling complex systems because they are structured to simulate individual behaviors and environmental conditions and have a natural spatial dimension [23, 24], all features that are not as easily captured in traditional mathematical models. In ABMs, the simulated population is made up of individuals (“agents”) that each have a unique set of characteristics and behave according to the rules defined in the model’s structure. This “bottom-up” structure allows for the modeler to easily manipulate the behaviors or the modeled environment and observe the emergent patterns that are produced by such manipulations. In the context of *T. solium* transmission, this structure facilitates application of the model to a variety of transmissions settings, and allows for testing a wide range of available control strategies, including spatially targeted strategies (e.g. “Ring Strategy” [25]), and other behavioral and structural interventions.

Our objectives in the analysis were to develop an ABM for *T. solium* transmission that included key spatial and behavioral features of *T. solium* transmission, and to subject the model to rigorous sensitivity analysis in order to identify sources of uncertainty in the model. In this article, we present the newly available model, called CystiAgent, with a detailed description of its structure and data sources, and results from rigorous sensitivity analysis applied to the model. The analyses were conducted with two major objectives in mind: (i) to evaluate the function of the model (i.e. is the model operating without error as it was designed?); and (ii) to investigate which parameters contribute most prominently to disease transmission, and consequently, have a high impact on uncertainty in the model outcomes. The first objective will provide quality assurance that the model is performing as expected, and the second objective will serve to prioritize a set of high-impact parameters for additional field studies to reduce uncertainty and account for variations between endemic regions.

## Methods

### Model description

CystiAgent is a spatially explicit ABM that is able to simulate endemic transmission of *T. solium* and test a variety of population-level interventions designed to control or eliminate *T. solium*. CystiAgent was developed in NetLogo 6.0.4 (Northwestern University, Evanston, IL, USA), an open-access ABM software that was chosen for its ability to represent spatial data and display simulations through a graphical interface. A basic version of the model, complete with the model code, graphical user interface, and supplemental data can be downloaded at http://modelingcommons.org/browse/one_model/6268. The model description adheres to the ODD (Overview, Design concepts, Details) protocol for describing ABMs [26].

#### Purpose

The purpose of CystiAgent is to deliver a model for *T. solium* that is able to accurately represent key spatial and behavioral aspects of transmission. This model structure has been designed with the flexibility to be applied to a variety of endemic settings and intervention types, which will facilitate validation against data from prospective trials, a key benchmark needed to test model accuracy. The ultimate objective of for CystiAgent is to have a model that can be used to compare available control and elimination strategies and provide evidence to support important policy decisions.

#### Entities, state variables, and scales

In CystiAgent, there are two agent classes—humans and pigs—that represent the definitive and intermediate hosts of *T. solium*, respectively. All humans and pigs are assigned to discrete household units that are distributed across a simulation village. Currently, CystiAgent is designed to simulate transmission in one village at a time (population up to ~ 2000), while all agents and processes are contained within the modeled village.

**Fig. 1:**
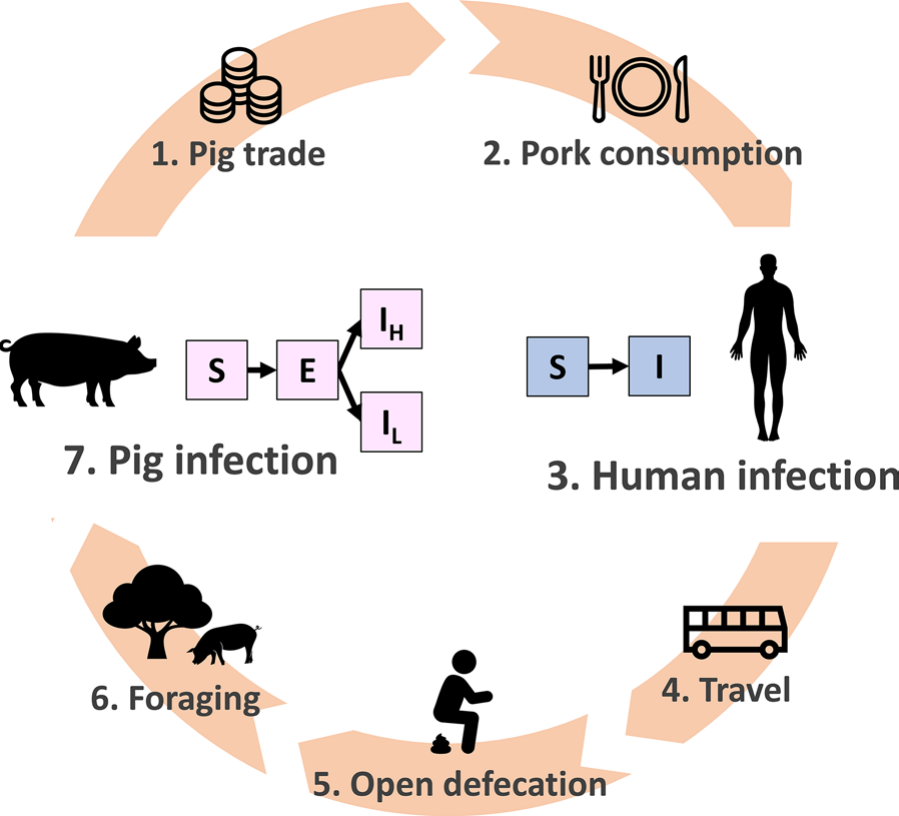
Transmission of *Taenia solium* in CystiAgent. (1) Pig trade: infected pigs are sold to other households inside and outside the village. (2) Pork consumption: infected pork meat is consumed by humans. (3) Human infection: susceptible humans (S) may be infected (I) with the adult-stage intestinal tapeworm through consuming heavily or lightly infected pork. (4) Travel: humans travel to other endemic villages where they may acquire tapeworm infections. (5) Open defecation: humans may practice open outdoor defecation. (6) Foraging: free-roaming pigs consume potentially infectious eggs present in the environment. (7) Pig infection: susceptible pigs (S) are exposed (E) to eggs or proglottids, and may become infected with heavy (I_H_) or light (I_L_) cyst infection

Each human and pig agent has an infection state, which is assigned at baseline and may change as they are exposed to infection risk throughout the simulation (Fig. 1). Humans may either be susceptible (S) or infected (I) with the adult-stage intestinal tapeworm (i.e. *T. solium* taeniasis). Human cysticercosis, including NCC or NCC-related seizure disorders, is not included in this model as it does not contribute to transmission.

Pigs may be either susceptible (S) or infected with metacestode larval-stages (i.e. porcine cysticercosis). Pig infection is categorized as heavy (≥ 100 cysts) (I_H_) or light (< 100 cysts) (I_L_) cyst burden, while pig exposure (E) includes the possibility of serological response to allow comparison with serological assays used in field studies. Cyst infection and serological response are assumed to be lifelong with no possibility of natural recovery or immunity, unless treatment or vaccine is applied.

Other state variables for humans and pigs are either assigned at the household or individual level. Household level variables include the x-y coordinates of the household, pig-raising by the household (yes/no), ownership of a pig corral (yes/no), use of the pig corral (always/sometimes/never), ownership of a latrine (yes/no), use of the latrine (always/never), the distance from the household that open defecation occurs when not using a latrine (log-normal distribution), and if a member of the household travels regularly outside the villages (yes/no). Human variables include whether an individual is a traveler (yes/no), and the frequency and duration of their travel to other endemic villages. There is no age or sex structure assigned to humans and there is no birth, death, or turnover of the human population. Individual pig variables include the current age of pigs (weeks), the age at which they will be slaughtered (weeks), the size of the roaming area (radius in meters), and if an individual pig is corralled at a given time (yes/no).

Each time-step of the model represents one week of cumulative activities and exposures. The one-week time step was the shortest period that could reasonably be achieved given computational limitations of the model while still providing enough accrued time for infections and other modeled behaviors to occur.

#### Process overview and scheduling

Model processes can be loosely categorized into seven steps that are depicted in Fig. 1.

#### Design concept: basic principles

CystiAgent consists of seven core functions that loop continuously in order to simulate natural endemic transmission:

1. *Pig trade.* Infected pigs that are due for slaughter may be butchered at home, sold within the village, or exported. Potentially infected pigs from external villages may also be imported into the village.
2. *Pork consumption.* Infected pigs are slaughtered by their owners and the resulting pork meat is either consumed at home or sold to other households, where it may cause human tapeworm infection.
3. *Human infection.* When consumed pork is infected with *T. solium* cysts, all members of the consuming households are exposed to potential tapeworm infection. If humans acquire a tapeworm infection, the intestinal tapeworm reaches maturity after 8 weeks [27, 28], and begins expelling infectious eggs at that time. Tapeworm infections naturally clear after pre-determined infectious durations [27, 28].
4. *Travel.* Humans that are designated as travelers leave the community at regular intervals, may contract tapeworm infections while traveling in other endemic areas, and return to the village after travel. Upon return, infected travelers resume contamination of their environment if applicable. Travel outside of the village is approximated in the model by sub-setting travelers and applying a different probability of infection without explicitly removing them from the simulation village.
5. *Open defecation.* Human tapeworm carriers that do not own or use a latrine release *T. solium* eggs and proglottid segments into the environment surrounding their household location. When tapeworm infections clear, humans stop releasing proglottid segments, but contamination of the environment with eggs persists until the eggs naturally degrade [29].
6. *Foraging.* Pigs that are designated as free-roaming (i.e. not contained in corrals) are exposed to *T. solium* proglottids and eggs that are present in their home-range areas.
7. *Pig infection*. Pigs that are exposed to proglottid segments may develop heavy cyst infection, while pigs exposed to eggs in the environment may develop light cyst infection. Either may result in seropositivity. Free-roaming pigs are exposed to an additional risk of infection or seropositivity that is proportional to the number of tapeworm carriers in the village and naïve to the pig’s location. This represents exposure to pigs that results from roaming and consumption of human feces from open defecation that occur outside of the home area.

#### Design concepts: interaction and stochasticity

Each model process above is defined mathematically by a corresponding parameter(s) that were derived from data collected in Peru or other literature sources (Table 1). Depending on the model activity they represent, most parameters correspond to the central value (e.g. mean) and spread (e.g. variance) of a chosen probability distribution. During setup and running of the model, continuous features are assigned to participants based on random number generation from the designated probability distribution, while categorical features and randomly assigned from a binomial distribution. As a result of the inherent stochasticity of each model parameter, model behavior varies considerably between each individual run, but predictable patterns emerge through repeated simulations.

**Table 1:** CystiAgent model parameters and plausible ranges used in sensitivity analyses

Parameter	Code	Distribution	Value	Plausible range		Source
				Lower	Upper	
Village setup						
Humans per household	humans-per-hh	Poisson	3.89	3.32	4.94	RST
Proportion of households raising pigs	prop-pig-owners	Binomial	0.49	0.25	0.75	
Pigs per pig-raising household	pigs-per-hh	Exponential	2.44	1.74	4.21	
Corral prevalence among pig-owner households	prop-corrals	Binomial	0.5	0.23	0.92	
Latrine prevalence	prop-latrines	Binomial	0.64	0.19	0.97	
Pig trade						
Pig slaughter age (median)	slaughter-age	Log-normal	9.8 months	9.5	10.0	RST
Proportion of pigs sold prior to slaughter	pigs-sold	Binomial	0.51	0.33	0.75	HH
Proportion of sold pigs exported	pigs-exported	Binomial	0.73	0.34	1	HH
Rate of pigs imported from endemic areas (imports/pig/week)	pig-import-rate	Uniform	0.00105	0	0.00384	HH
Prevalence of cyst infection among imports	import-prev	Binomial	0.134	0	0.3	[18]
Proportion of infected imported pigs with light cyst burden	light-to-heavy	Binomial	0.76	0.5	1	[18]
Pork consumption						
Proportion of pork consumed by owner	hh-only-pork	Binomial	0.40	0.22	0.71	HH
Proportion of pork sold after slaughter	sold-pork	Binomial	0.12	0	0.5	HH
Proportion of shared pork eaten by owner	shared-pork-hh	Binomial	0.8	0	0.84	HH
Human infection						
Incubation time to reach tapeworm maturity	tn-incubation	Fixed	8 weeks	-	-	[27, 28]
Tapeworm lifespan (mean, SD = 1 year)	tn-lifespan	Normal	2 years	0.5	4	
Travel						
Proportion of households with a frequent traveler	traveler-prop	Binomial	0.42	0.24	0.65	HH
Frequency of travel to other endemic areas (every × weeks)	travel-freq	Uniform	8 weeks	5	16	HH
Duration of travel	travel-duration	Exponential	1.75 weeks	0.84	3.36	HH
Incidence of *T. solium* taeniasis during travel (risk/person/week)	travel-incidence	Uniform	0.00023	0.00004	0.002	[18]
Open defecation						
Latrine-use (prop. of households that “always” use latrine)	latrine-use	Binomial	0.25	0	0.86	GPS
Radius of environmental contamination (median, meters from home)	cont-radius	Log-normal	26 meters	23	30	GPS
Rate of egg decay in environment (mean survival duration)	decay-mean	Exponential	8 weeks	1	26	[29]
Foraging						
Proportion of pig households with corrals that “always” corral pigs	corral-always	Binomial	0.05	0	0.39	GPS
Proportion of pig households with corrals that “sometimes” corral pigs	corral-sometimes	Binomial	0.57	0.25	0.61	
Proportion of pigs in “sometimes”-corral-households that are corralled	prop-corral-some	Binomial	0.32	0.15	0.44	
Radius of pig roaming “home-range” (median)	home-range	Log-normal	44 meters	30	96	
Tuning parameters^a^						
Probability of human taeniasis upon slaughter of lightly infected pig	pl2h	Binomial	0.0163	0.03	0.4	CAL
Probability of human taeniasis upon slaughter of heavily infected pig	ph2h	Binomial	0.0046	0.003	0.04	
Probability of light cyst infection upon contact with *T. solium* eggs	light-inf	Binomial	0.0074	0.003	0.02	
Probability of heavy cyst infection upon contact with *T. solium* proglottids	heavy-inf	Binomial	0.0059	0.003	0.02	
Probability of exposure to *T. solium* eggs per human with taeniasis^b^	light-all	Binomial	0.0141	0	0.05	
Probability of exposure to *T. solium* proglottids per human with taeniasis^b^	heavy-all	Binomial	0.0182	0	0.05	

^a^Listed values for tuning parameters were calibrated to a single test village used to assess baseline model function in Fig. 2

^b^Exposure probabilities (“light-all” and “heavy-all”, *x*) scaled to the current number of tapeworm carriers (HT) according to 1 − (1–*x*)^HT^

*Abbreviations*: HH, Household survey; GPS, GPS pig tracking study, RST, Ring Strategy; CAL, calibration; SD, standard deviation

#### Design concepts: emergence and observation

The emergent outputs of CystiAgent are the prevalence of human taenaisis and the prevalence of porcine cysticercosis, which includes the prevalence of pigs with heavy and light infection burdens, and pigs that are seropositive. These outputs are recorded at each weekly time step.

#### Design concepts: collectives

Since pigs and humans belong to households that share traits and a spatial environment, clustering of behaviors and emergent patterns of infection occur among pigs and humans in the same households, and among households that are in close proximity.

#### Design concepts: other

The agents in CystiAgent do not have adaptive traits, or the ability to learn from or sense features of their environment. Behaviors are determined strictly by the parameters and state variables that are defined at the initialization of a model run.

#### Input data

A variety of sources, including primary data, literature review, and expert opinion, were utilized to determine the values and distributions for model parameters. For the majority of parameters, we used data collected in the Piura region of northern Peru. A full description of the methods and data sources used to estimate each parameter value can be found in Additional file 1: Text S1. For the purposes of sensitivity analyses, we designated a “plausible range” of values for each parameter in addition to its estimated central value. This is a range of values across which the model was evaluated to determine the impact of each parameter on model outputs. In some cases, the plausible range was determined by adopting the range of mean values observed across a group of endemic villages, and in other cases we manually widened the range to account for additional uncertainty and variability in the parameter.

For six parameters that could not be determined through primary data collection or experimentation, we estimated their values using an approximated Bayesian computation (ABC) algorithm [30]. These parameters (which will be referred to as “tuning parameters”) include two that define the probabilities of tapeworm infection after slaughter of heavily (“ph2h”) and lightly (“pl2h”) infected pigs; two that define the probability of heavy and light pig infection after exposure to proglottid segments (“heavy-inf”), and eggs (“light-inf”) present in the environment; and two that determine the probability of exposure to proglottid segments (“heavy-all”) or eggs (“light-all”) during pig-roaming outside of a pig’s home-range area.

#### Initialization

The NetLogo spatial environment is populated by assigning an x-y coordinate to each household in a village (these can be based on real or fictitious villages). Pigs and humans are then assigned to households based on the population characteristics of the village, which can be done with census data from a real village or other data sources. State variables, including infection status, are randomly assigned to humans and pigs based on the probabilities defined by corresponding model parameters. The prevalence of human taeniasis and porcine cysticercosis at baseline may be set by the user, or set to level observed in a given dataset. Once the model begins to run, however, the prevalence levels will stabilize at a natural endemic equilibrium. CystiAgent utilizes the six tuning parameters described above to adjust transmission levels in the model to a desired level in a given village. Calibration of these tuning parameters is not a required step, but would be needed for validation of the model against a specific observed dataset.

The ABC method adapted for CystiAgent tuning follows a simple “rejection sampling” approach and is based on a variety of in-depth examples found in literature [31–33]. Briefly, random values are sampled from a uniform distribution for each of the tuning parameters, and each combination of parameter values is run in the model without varying other model parameters. The average prevalence of human taeniasis and porcine cysticercosis are measured for each run and the Euclidean distance between these values and the target prevalence levels are calculated. Following a rejection sampling scheme, we select the top 1% of model runs that minimized the Euclidean distance and extract posterior distributions from the selected parameter sets. We then repeat the algorithm until a final set of parameter values is produced that adequately replicates the target prevalence levels.

#### Intervention tools

CystiAgent has the ability to simulate a variety of population-level interventions designed to control or eliminate *T. solium* transmission. A generic function is available to administer anthelminthic treatment (e.g. niclosamide) for human taeniasis, either presumptively or after stool screening. Other functions include the treatment of pigs to cure cystic larval infection (e.g. oxfendazole), or vaccination to prevent infection. For each intervention type, user-controlled options allow for specification of participation levels, the sensitivity of screening tests, and the efficacy of drugs and vaccines used. These interventions can then be implemented through mass or targeted approaches, while varying the duration and frequency of intervention applications. Unique to this spatial model is the ability to simulate spatially targeted interventions. “Ring strategy” [25] can be applied by targeting treatment resources to households residing within a given distance of heavily infected pigs. Finally, behavioral and structural interventions such as improved access to corrals and latrines are available as stand-alone interventions or in combination with other approaches. While available in the model, not all intervention types were applied or evaluated in the present analysis.

### Baseline model function and intervention application

In order to examine the stability and functionality of CystiAgent, we set up the model with observed data from Peru and applied three unique test scenarios: endemic equilibrium (no intervention), combined ring treatment strategy, and combined mass treatment strategy. The test village we used for these simulations is an endemic village in the northern Peruvian region of Piura that recently participated in a prospective trial testing a variety of *T. solium* control strategies (SEO, unpublished data). Household coordinates, input population characteristics, and prevalence of human taeniasis and porcine cysticercosis were estimated at baseline in the parent study and were made available for use in the model by the study authors.

To apply the test scenarios to CystiAgent, we first used the ABC algorithm to calibrate the model’s tuning parameter to observed transmission levels in the village, and then ran each of the scenarios across 500 Monte Carlo simulations. The first scenario (no intervention) consisted of 300 weeks without intervention. In the second scenario (combined ring treatment), we applied seven rounds of a combined human and porcine ring treatment over a two-year period. This included screening pigs for infection using the tongue inspection method every four months, and offering treatment to all human and pigs that resided within 100 meters of the identified pig. In the third scenario (combined mass treatment), all humans and pigs were offered treatment, which was applied every six months for a total of five rounds. Details of each intervention application, including drug efficacy and treatment coverage for humans and pigs are listed in the figure caption.

### Sensitivity analysis of CystiAgent

#### Process overview

We performed all sensitivity analyses in R version 3.5.1, using the *RNetLogo* package [34] to execute model simulations in NetLogo from R. Sensitivity analyses included the Latin hypercube sampling partial rank correlation coefficients (LHS-PRCC) and Sobol’ variance decomposition. Only the results of the LHS-PRCC will be presented here, however, as results were similar between the two methods. A description of the Sobol’ method and results are available in Additional file 2: Text S2 and Additional file 2: Figure S1). Both methods were applied in three unique villages with different population sizes and housing densities. Household coordinates for the three test villages were based on real endemic villages in northern Peru that recently participated in a large prospective trial (SEO, unpublished data). For evaluation of the CystiAgent model, sensitivity analyses were applied to two model versions: the crude model in which all parameters (*k* = 33) were evaluated for their impact on model outcomes, and a calibrated model for which village input characteristics and tuning parameters were fixed so that a smaller set of biological and behavioral parameters (*k* = 22) could be evaluated. For the calibrated model, fixed values for village input characteristics (i.e. humans and pigs per household, pig ownership, corral and latrine access) were based on data from the census applied in each village, while tuning parameters were estimated using the ABC algorithm [30], described above, to fit the model to observed levels of human taeniasis and porcine cysticercosis in each village. Each run of the model in sensitivity analyses consisted of 1000 weeks of stable endemic transmission with no interventions applied. The summary statistics collected at the end of each run were defined as the incidence-density of human taeniasis (number of new infections/100 person-years), and the lifetime cumulative incidence of porcine cysticercosis (cumulative number of infected pigs/cumulative pig population).

In order to achieve the computational resources needed to run the model through many thousands of simulations for each of these analyses, we executed all model simulations on the Amazon Web Service EC2 cloud computing platform. Model simulations were distributed across a 72-core parallel processor using the *parallel* R-package [35] and executed on the EC2 cloud using the R-Studio Shiny server [36].

#### Latin hypercube sampling-partial rank correlation coefficient (LHS-PRCC)

A detailed description of LHS-PRCC method can be found elsewhere [37]. Briefly, LHS-PRCC provides a non-parametric measure of the strength of monotonic association between each parameter and each outcome of the model (human taeniasis and porcine cysticercosis incidence). For application of LHS-PRCC, we first determined the plausible ranges for each model parameter as described above, and sampled values from each parameter distribution using a Latin hypercube sample. This procedure involves dividing each parameter range into *n* equal segments, and selecting a random value from each segment, as described [38]. For LHS-PRCC analyses on both the crude (*k* = 33 parameters) and calibrated (*k* = 22 parameters) models, we chose equivalent sample sizes (*n*) of 175,000, 50,000 and 50,000 for low, medium, and high-density villages, respectively. We then ran the model through all parameter permutations and analyzed the results to determine partial-rank correlation coefficients for each parameter using the *sensitivity* and *ppcor* R packages. For this, the PRCC formula calculates the linear correlation, *ρ*, between the residuals of the rank-transformed parameter input and rank-transformed model output, while accounting for correlations with all other parameter inputs [37]. Importantly, the final PRCC estimates provide measures of the strength, direction, and statistical significance of the association between parameter inputs and model outputs. *P*-values were obtained with a Student’s t-distribution and were evaluated with a Bonferroni adjustment for 33 multiple comparisons (*P* < 0.0015 for statistical significance).

## Results

### Baseline model function and intervention application

**Fig. 2:**
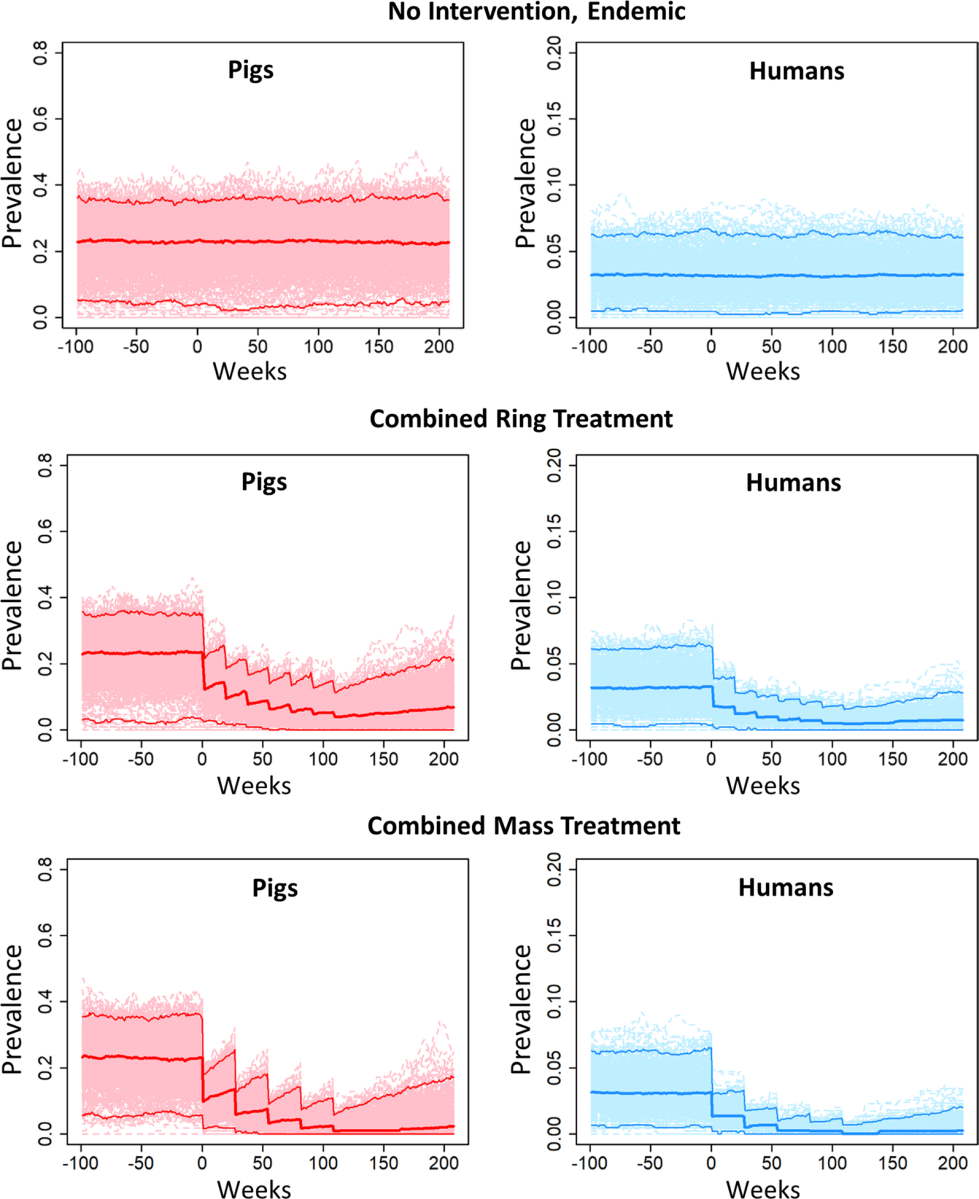
Example of model outcomes in three test scenarios. Scenarios tested were endemic equilibrium (top), combined ring treatment (middle), and combined mass treatment (bottom), and outcomes shown are porcine cysticercosis (left) and human taeniasis (right). For each scenario the model was calibrated to a baseline prevalence 23.2% for porcine cysticercosis and 2.9% for human taeniasis. Intervention settings for ring treatment: 87% treatment efficacy for human taeniasis (2-dose niclosamide); 100% treatment efficacy for porcine cysticercosis (oxfendazole); 91% sensitivity of tongue inspection; 85% participation among humans in rings, 60% pig participation. Intervention settings for mass treatment: 77% treatment efficacy for human taeniasis (1-dose niclosamide); 100% treatment efficacy for porcine cysticercosis (oxfendazole); 75% participation among all humans; 60% participation among all pigs

When the model was calibrated with data from an endemic village in Peru and run at endemic equilibrium, the median simulated prevalence of porcine cysticercosis was 23.3% (Fig. 2). This was consistent with the target prevalence of 23.2%, which was estimated from the baseline data for the test village in the Ring Strategy Trial. The median simulated prevalence of human taeniasis at baseline was 3.2%, which is slightly higher than the 2.9% prevalence estimated from the test village in the trial.

When the ring treatment intervention and mass treatment interventions were applied to the simulation village, each demonstrated a significant reduction in the prevalence of human taeniasis and porcine cysticercosis. Neither strategy consistently achieved elimination of transmission, and a rebound in transmission was observed after the final round of treatment.

### Sensitivity analysis: crude model

**Fig. 3:**
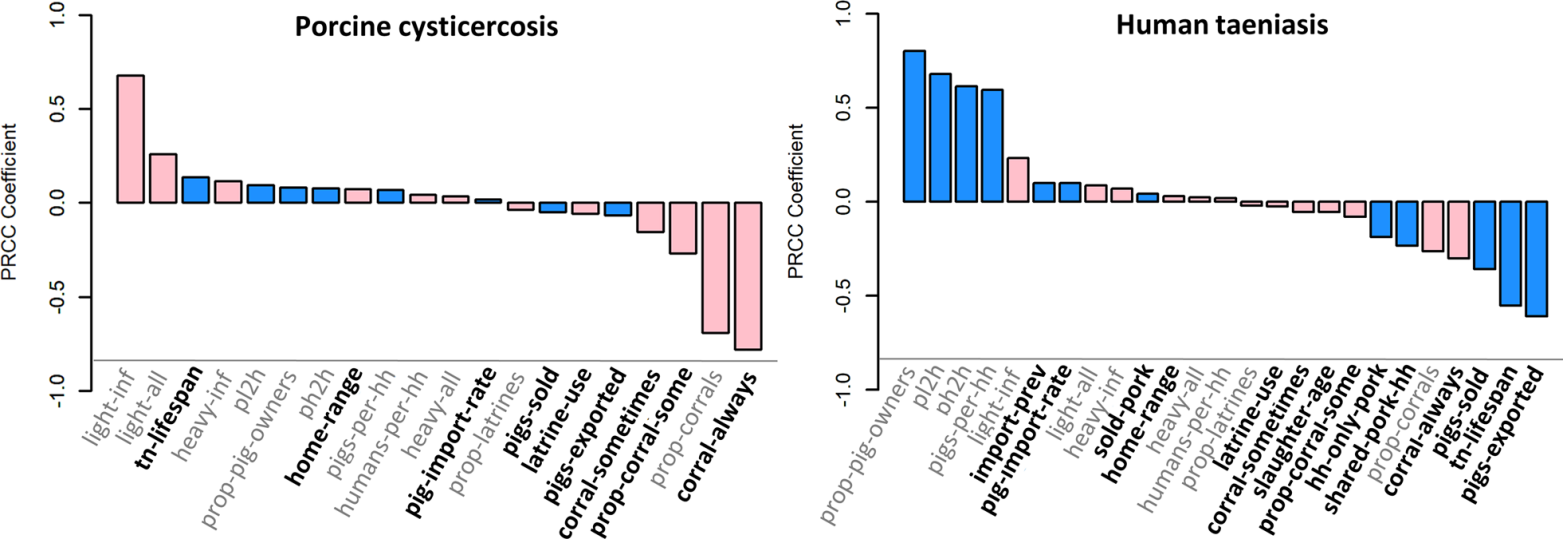
Partial rank correlation coefficients for porcine cysticercosis (left) and human taeniasis (right) in the crude model, medium-density village. Bar colors represent the primary impact of each parameter (blue, human taeniasis; pink, porcine cysticercosis). Parameters in grey (six tuning parameters and five village setup characteristics) were not evaluated in the calibrated model. Only parameters with *P*-values < 0.0015 are shown. See Table 1 for a description of parameter names and functions

Sensitivity analysis of the crude CystiAgent model with LHS-PRCC identified a similar set of highly influential parameters across all three villages tested (low, medium and high density). Of the 33 parameters included, those with the greatest impact on porcine cysticercosis as a model outcome were the parameters defining the use of corrals to contain pigs, and pig-related tuning parameters. Most prominently, this included the proportion of pig-owners that own a corral (“prop-corrals”), “always” corral their pigs (“corral-always”), or sometimes corral their pigs (“corral-sometimes” and “prop-corral-sometimes”), which were all highly protective for pigs across all three villages tested. Pig-related tuning parameters were also highly impactful on pig infection in the crude model. These included the probability of light cyst infection after exposure to environmental egg contamination (“light-inf”) and the probability of exposure to environmental egg contamination outside of home-range (“light-all”). Figure 3 shows LHS-PRCC coefficients from the analysis of the crude model on the medium-density village, while the results from all three village are presented in Additional file 3: Figure S2.

For human taeniasis as the model outcome, the four parameters most strongly correlated with increased incidence were the two human-related tuning parameters (“pl2h” and “ph2h”), the proportion of households that raise pigs (“prop-pig-owners”), and the mean number of pigs per household (“pigs-per-hh”). Parameters that were strongly associated with a decreased incidence of taeniasis in all three villages included the export of pigs out of the village (“pigs-exported”), the sale of pigs prior to slaughter (“pigs-sold”), and an increased duration of tapeworm infection (“tn-lifespan”). In addition to these strong correlations, the rate of pig import (“pig-import-rate”) and the prevalence of cyst infection among imported pigs (“import-prev”) were consistently correlated with small increases in taeniasis incidence, while parameters that promoted consumption of pork at home (“hh-only-pork”, ”shared-pork-hh”) were associated with small decreases in taeniasis incidence.

### Sensitivity analysis: calibrated model

**Fig. 4:**
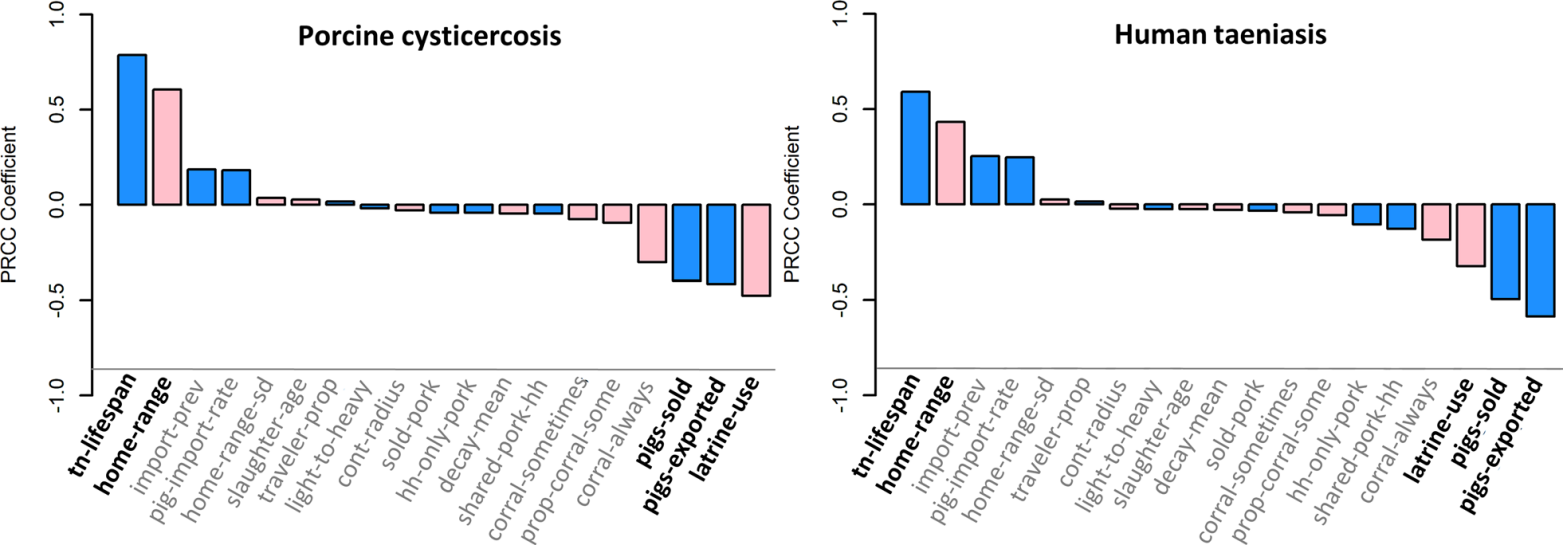
Partial rank correlation coefficients for porcine cysticercosis (left) and human taeniasis (right) in the calibrated model, medium-density village. Bar colors represent the primary impact of each parameter (blue, human taeniasis; pink, porcine cysticercosis). Parameters in bold (“tn-lifespan”, “home-range”, “pigs-sold”, “pigs-exported”, and “latrine-use” represent the five most impactful parameters for both outcomes). Only parameters with *P*-values < 0.0015 are shown. See Table 1 for a description of parameter names and functions

When tuning parameters and village characteristics were fixed for the analysis of the calibrated model, the set of parameters that impacted transmission changed considerably (Fig. 4). Of the 22 parameters included in this analysis, the most consistently impactful parameter for both porcine cysticercosis and human taeniasis was the average duration of taeniasis (“tn-lifespan”), which had measured correlation coefficients of *ρ* = 0.63, 0.79 and 0.71 for porcine cysticercosis and *ρ* = 0.49, 0.59 and 0.57 for human taeniasis in the low, medium and high-density villages, respectively. In addition to tapeworm lifespan, the size of pig home-ranges (“home-range”), the rate of pig import (“pig-import-rate”) and the prevalence of cyst infection among imported pigs (“import-prev”) were all significantly correlated with increased incidences of porcine cysticercosis and human taeniasis in all three villages; while the use of latrines (“latrine-use”), proportion of pigs exported (“pigs-exported”), proportion of pigs sold (“pigs-sold”), and use of corrals to contain pigs (“corral-always”) were all significantly correlated with reduced rates of both porcine cysticercosis and human taeniasis in all three villages.

## Discussion

The primary objective of this research was to develop a functional ABM capable of simulating the complex behavioral, biological, and environmental factors that contribute to *T. solium* transmission in endemic areas. Our evaluation of baseline model function demonstrated that the CystiAgent model can be accurately tuned to a desired level of transmission in a given village. While this demonstration only consisted of a single village in northern Peru, we have tested CystiAgent on over 40 unique villages in Peru, and the model has consistently replicated observed transmission levels. To achieve the wide range of prevalence levels seen across endemic villages, calibrated values for tuning parameters may vary considerably between villages, and calibration is not always successful. Smaller villages (< 75 households) and those with human taeniasis prevalence < 1% or porcine cysticercosis < 10% have been less likely to achieve stable endemic equilibrium after calibration.

Application of two unique intervention strategies in CystiAgent demonstrates that the model is able to simulate a variety of intervention strategies, including mass-applied and spatially-targeted interventions. The ability to calibrate transmission levels to specific endemic levels and simulate a variety of intervention types will allow for validation of CystiAgent against data from prospective trials, a step that will be needed to test the accuracy of future model predictions. The present analysis allows us to move closer to this goal by both demonstrating the ability of the CystiAgent model to represent the complex dynamics of *T. solium* transmission, and identifying key model parameters that must be investigated in order to apply the model to specific endemic settings in the future.

In our sensitivity analysis of the crude model, we found that the parameters that had the strongest impact on model variability were the “tuning” parameters that defined probabilities of infection in the model. For porcine cysticercosis, these included the probabilities of heavy or light infection upon contact with *T. solium* eggs or proglottids in the environment, and for humans, these included the probabilities of tapeworm infection upon consumption of heavily or lightly infected pork. Due to their considerable impact on transmission in the model, and the wide range of values they can assume, statistical calibration of the values of these parameters is highly recommended for application of the model to any specific transmission setting. Approximated Bayesian computation [30], which was the method we chose to employ, or other available parameter estimation methods [33], can be used for this purpose. At a minimum, this process would require knowledge of the prevalence of human taeniasis and porcine cysticercosis in the targeted population, but could be improved if additional local population characteristics and behavioral parameters were known for the target population.

Apart from these tuning parameters, many of the highly impactful parameters identified in our analysis of the crude model fell into the category of village characteristics. These were parameters that defined the number of households raising pigs, the number of pigs per household, and access to corrals to contain pigs. The impact of these parameters on transmission levels demonstrates the importance of local variation in population structure and pig-raising practices on *T. solium* transmission dynamics. In light of their impact, determining local values for these village characteristics should be a priority when applying the model to specific endemic settings. Steps such as population census or consultation with local leaders to acquire information about the size and characteristics of the pig and human populations would allow for reduced uncertainty and improved model accuracy.

We conducted our analysis of the calibrated model in order to see beyond the tuning parameters and village characteristics that were driving uncertainty in our crude model. This analysis allowed us to assess the impacts of a smaller set of biological and behavioral parameters in the context of transmission levels that were tuned to more realistic levels. In the calibrated model, the average duration of tapeworm infections (“tn-lifespan”) emerged as the most significant source of uncertainty in all villages and analyses. The size of pig home ranges (“home-range”), the proportion of households that regularly use latrines (“latrine-use”), and the sale (“pigs-sold”) and export (“pigs-exported”) of pigs were also consistently identified as impactful in the calibrated model.

The impacts attributed to parameters in the calibrated analysis reflect both the strength of the relationship they have with model outputs, and the amount of uncertainty defined in the parameter values themselves (i.e. the width of the defined “plausible range”), which exerts considerable leverage on a parameter’s measured impact. Each of the key parameters identified above were varied across wide ranges due to our uncertainty in the true value of the parameter (e.g. mean tapeworm lifespan ranged from 6 months to 2 years, the percent pigs exported ranged from 34% to 100%, etc.; see Table 1).

For biological parameters like tapeworm lifespan, this high degree of uncertainty is due to limited knowledge from experimental studies [27, 39], and data is unlikely to improve due to ethical constraints on experimental tapeworm infection. For other parameters, wide uncertainty ranges are due to the variability that exists between endemic villages and regions. Each of these factors depends on cultural, behavioral, and economic practices that are context-specific. For example, estimates for the home ranges of free-roaming pigs were based on a GPS study recently completed in three villages of northern Peru [40], but even within this restricted locale, variations in topography, landscape, and pig management led to substantial differences between villages. Similar between-village variations were seen in the sale and export of pigs, which served as a primary economic activity in some rural villages evaluated, and a rare source of emergency income in others. Finally, the prevalence and use of latrines varied considerably between villages depending on whether state-sponsored latrine construction had been implemented in the village. Taken together, these local variations are important to take into account when applying the model to specific endemic settings. As with key village characteristics outlined above, investigation of these local behavioral features through surveys or expert consultation prior to application of the model would reduce parameter uncertainty and likely improve validity of the model for that setting.

The parameters identified as impactful in our sensitivity analyses are generally consistent with the only other published sensitivity analysis for a *T. solium* transmission model [13]. The EPICYST model is a deterministic mathematical model that includes human cysticercosis as a primary model outcome and was parameterized based on data from *T. solium* transmission in a sub-Saharan Africa. Consistent with our findings, an LHS-PRCC analysis of EPICYST revealed the most influential parameters to be “transmission coefficients” that define the rates of infection upon exposure, the expected duration of tapeworm infections, and the rate of pork consumption among humans. Since EPICYST is a population-level model and does not include individual behaviors or a spatial framework, it was not able to provide a comparison to other important features of our model such as pig corralling, pig roaming, and latrine use.

There are a few important strengths and limitations of our approach to highlight. First, we chose to design CystiAgent within the framework of an ABM, which allowed us to account for the complex spatial and behavioral heterogeneities that affect *T. solium* transmission in endemic areas. Despite this strength, CystiAgent only begins to account for the complex heterogeneities that likely occur in real-world systems. Age-related differences in pig roaming patterns [20], seasonal and climate-related variations in transmission [41], acquired immunity and resistance among pigs [42], vector-borne transmission of *T. solium* eggs to pigs [43, 44], and black-market distribution of infected pork [45] are only a few of the many additional factors that may impact transmission patterns and are not explicitly defined in CystiAgent. Additional data from experimental or field studies and will be needed in order to incorporate these features into future versions of the model and evaluate their impact on transmission.

Secondly, the parameter inputs used in CystiAgent were primarily sourced from a single region of northern Peru through extensive work conducted in the region over the past decade. The depth of data available in this region is a strength of our approach and made it possible to construct this detailed ABM. Nonetheless, parameter values that are accurate for this region of Peru may be vastly different from corresponding settings in other endemic regions due to environmental and cultural factors. Application of the model to new regions would likely require some level of input data for key parameters alongside and local calibration of tuning parameters. Since this degree of detailed behavioral and environmental data may not be available in areas of the world with more limited research infrastructure, the model may have to be adapted or simplified to function in these settings, which could limit the generalizability of the model in its current form. That said, the results of our sensitivity analyses showed that model outputs are robust to variations in all but most sensitive parameters.

Finally, an important strength of our sensitivity analyses was our use of two complementary methods (Sobol’ and LHS-PRCC) and our application of the methods on three villages of differing population sizes and densities. The consistency of our results between methods and villages provides confidence that the key features of the model are robust to variation in population structure and methodology. Despite these promising findings, the model could be tested in additional endemic settings to provide further insight into parameter relationships. Perhaps most importantly, sensitivity analyses should be conducted in the context of control interventions, as key parameters that affect transmission at endemic equilibrium (e.g. human travel and migration, tuning parameters that approximate probabilities of infection given exposure) may be different when control pressure is applied.

## Conclusions

In this research, we developed a functional ABM that is able to represent the core features *T. solium* transmission observed in endemic settings. Our replication of baseline model function and application of control interventions demonstrated that the CystiAgent model functioned as expected and was able to be tuned to specific prevalence levels observed in endemic village. Despite significant uncertainty in some key model parameters, the robustness of our model to variations in all but the most sensitive parameters suggests that the model is likely to be transportable to other endemic settings outside of Peru, given local specification of these key parameters and calibration of tuning parameters to local levels of transmission. While the generalizability of the model to other populations outside of Peru will remain unknown until it is tested in these settings, we have conducted validation of CystiAgent model against data from prospective trials conducted in Peru, and will present the results of this validation in a future publication. Ultimately, our goal is to provide this validated model as a tool for researchers and policy-makers seeking to compare available control strategies for *T. solium* and prioritize promising strategies for evaluation in prospective trials.

## Supplementary information

**Additional file 1: Text S1.** Data sources and statistical methods for CystiAgent parameters.

**Additional file 2: Text S2.** Supplemental methods and results for Sobol’ variance decomposition. **Figure S1.** Graphical results of Sobol’ variance decomposition for crude and calibrated model versions on the medium-density village.

**Additional file 3: Figure S2.** Latin hypercube sampling-partial rank correlation coefficient (LHS-PRCC) results of crude and calibrated models across low, medium, and high-density villages. Parameters with significant LHS-PRCC coefficients (*P* < 0.0015) are shown.

## Data Availability

The data collected for this study are available from the corresponding author upon request.

## Abbreviations

ABM: Agent-based model
LHS-PRCC: Latin Hypercube Sampling-Partial Rank Correlation Coefficient
WHO: World Health Organization
