# Supplementary material for: Understanding transmission and control of the pork tapeworm with CystiAgent: a spatially explicit agent-based model
Source: Parasit Vectors. 2020 Jul 24;13:372. doi: 10.1186/s13071-020-04226-8 (PMC7379812; doi:10.1186/s13071-020-04226-8)
Supplement: Supplementary file 1 — Additional file 1: Text S1. Data sources and statistical methods for CystiAgent parameters. [file 13071_2020_4226_MOESM1_ESM.pdf]

Additional file 1. Text S1. Data sources and statistical methods for CystiAgent parameters

SOURCE #1: Ring Strategy Trial (RST)

Model parameters:

humans-per-hh	mean number of people assigned to each household
prop-pig-owners	proportion of households that raise pigs
pigs-per-hh	mean number of pigs assigned to each pig-raising household
prop-corrals	proportion of pig-raising households that own corrals for their pigs
prop-latrines	proportion of households that have access to a latrine
slaughter-age	mean age at which pigs are slaughtered

Description of study:
This was a large cluster-randomized trial comparing the effectiveness of "Ring-strategy" versus mass-treatment as control strategies to reduced *Taenia solium* transmission. The trial was carried out in 23 rural villages of the northern Peruvian region of Piura between 2015-2017. Data from this study that were used in the CystiAgent model included the baseline census, which collected demographic variables for all households in the study, and serial follow-up of cohorts of pigs, which were captured every 4 months to collect serum samples. The RST study was funded by NIH grant number NIH R01-NS080645 with Seth O'Neal as principle investigator.

Methods/results:
Village input parameters. A household-level census was conducted at baseline in all 23 study villages, and attempted to gather information on all residents of the study villages. Variables recorded for the census included demographics of all human inhabitants, the condition of the house, access to water and sanitation, and livestock including pigs. For each variable of interest, results were summarized by village. The plausible ranges for parameters were determined by the maximum and minimum means observed across all villages. Probability distributions for discrete parameters (*humans-per-hh, pigs-per-hh)* were determined by fitting raw census data to a range of distributions (e.g., normal, log-normal, Poisson, exponential) and selecting the distribution with the best fit based on AIC values (Akaike Information Criterion). The Poisson and exponential distributions chosen to represent *humans-per-hh* and *pigs-per-hh*, respectively are defined by a single mean value, and were truncated at 1 to prevent household with 0 inhabitants.

Parameter	Distribution	Mean	Lower	Upper
humans-per-hh	Poisson	3.89	3.32	4.94
prop-pig-owners	Binomial	0.49	0.25	0.75
pigs-per-hh	Exponential	2.44	1.74	4.21
prop-corrals	Binomial	0.5	0.23	0.92
prop-latrines	Binomial	0.64	0.19	0.97

slaughter-age: The age of pigs at slaughter was estimated for two cohorts of study pigs (n=1,284 pigs) that entered the study at baseline and month 4 (M4), when they were between 2-4 months old (this is when age estimation is most accurate). Pigs were captured for serum-sampling every 4 months throughout the study. Therefore, when a pig from one of these cohorts was censored from the study (not captured in the following sample), its age at censorship (i.e., slaughter) was assumed to be the age it would have reached at the mid-point of the previous 4-month interval. Pigs still alive at the end of the study (n=47 pigs) were conservatively given slaughter-ages of their age in the last sampling round plus two additional months. Final slaughter ages were analyzed to determine the probability distribution and summary statistics to best describe them. A log-normal distribution was chosen as the optimal fit, and the

log-mean and log-standard-deviation were estimated from the data. The "plausible range" for log-mean was derived from a 95% confidence interval produced through bootstrapped resampling (n=1000).

**Slaughter-age to follow a LOG-NORMAL distribution**			
	**Value**	**Lower**	**Upper**
Log-mean	2.279	2.249	2.305
Log-SD	0.515	-	-

SOURCE #2: Household survey (HH)

Model parameters:

pigs-sold	Proportion of pigs sold prior to slaughter
pigs-exported	Proportion of sold pigs that are exported to other villages
pig-import-rate	Rate of pigs imported from other endemic villages (import / pig / week)
hh-only-pork	Proportion of slaughtered pigs that are consumed exclusively by the owner's household
sold-pork	Proportion of slaughtered pigs that are exclusively sold to another household in the same village
shared-pork-hh	Among slaughtered pigs shared between the owner and other households, the proportion pork-meat eaten by the owner's household
traveler-prop	Proportion of households that have a member who regularly travels
travel-freq	Interval of time (in weeks) between trips to other endemic villages
travel-duration	Average duration of trips to other endemic villages

Description of study:
The "household survey" was a door-to-door survey applied in 7 rural villages of northern Peru (Piura region) between 2017-18. The survey was applied to all heads-of-household that resided in the study villages, and was carried out over two time-points four months apart (n1=420 and n2=410 households). The survey was applied as part of a community-based study that aimed to identify methods for improving reporting of infected pigs to the health post. Survey questions used for the CystiAgent model were embedded in a larger survey that assessed knowledge, behaviors, and attitudes towards cysticercosis. The parent study was funded by NIH grant number NIH R01-NS080645 with Seth O'Neal as principle investigator.

Methods/results:
pigs-sold: The survey question asked (translated from Spanish): "How many pigs from your household have you sold prior to slaughter in the past 4 months?" The total number of pigs sold in each village was divided by the total number of pigs slaughter *or* sold in the same interval to determine the proportion of pigs sold among those due for sale or slaughter. The final parameter value was determined by averaging the results of the two survey time-points. The "plausible range" was determined by selecting the minimum and maximum values from among the seven villages surveyed.

	**Estimate**	**LL**	**UL**
Total pigs sacrificed or sold	760		
**Pigs SOLD**	51.4% (391/760)	32.6%	75.4%

pigs-exported: The survey question asked (translated from Spanish): "How many pigs from your households have you sold prior to slaughter *outside of the village* in the past 4 months?" The total number of pigs sold *outside of the village* (i.e., exported) was divided by the total number of pigs sold to determine the proportion of sold pigs that were exported. The final parameter value was determined by

averaging the results of the two survey time-points. The "plausible range" was determined by selecting the minimum and maximum values from among the seven villages surveyed.

	Estimate	LL	UL
Total pigs sold	391		
**Pigs EXPORTED**	73.1% (286/391)	34.2%	100%

<u>*pig-import-rate:*</u> The survey question asked (translated from Spanish): "How many live pigs have your purchased from *outside the village* in the past 4 months?" The total number of pigs imported was divided by the total number of pigs in the village and the 4-month period (17 weeks) to determine the number of pigs imported per pig in the population per week. The final parameter value was determined by averaging the results of the two survey time-points. The "plausible range" was determined by selecting the minimum and maximum values from among the seven villages surveyed.

**Parameter**	**Estimate**	**LL**	**UL**
Total pigs in the village	1956		
Total pigs purchased externally	35		
**IMPORTED pigs (per pig per week)**	0.00105	0	0.00384

<u>*hh-only-pork, sold-pork:*</u> The survey questions asked (translated from Spanish): "How many pigs from your households have you slaughtered in the past 4 months?" and, among those, "How many were consumed exclusively by members of your household?", "How many were sold or gifted after slaughter to other households?" and "How many were shared between members of your household and other households?" The proportion of pigs eaten at home, sold, and shared were calculated by dividing each total by the total number of pigs slaughtered. The proportion shared is not explicitly defined as a model parameter because it is represented by the proportion of pigs that remain after the first two parameters are applied. The final parameter value was determined by averaging the results of the two survey time-points. The "plausible range" was determined by selecting the minimum and maximum values from among the seven villages surveyed.

**Parameter**	**Estimate**	**LL**	**UL**
Total pigs slaughtered by household	366		
**Pigs consumed exclusively by HOUSEHOLD**	39.6% (145 / 366)	21.7%	71.4%
**Pigs exclusively SOLD to other households in village**	11.5% (42 / 366)	0%	50.0%
**Pigs SHARED between household and other households in village**	48.9% (179 / 366)	12.5%	75.0%

<u>*shared-pork-hh:*</u> The survey question asked (translated from Spanish): "How many kilos of pork meat have you purchased from within your village in the past 4 months?" This total was divided by the total number of kilos of pork meat shared or sold within the village, which was determined taking the total number of pigs sold or shared in the village and applying an average of 50 kg per pig times 0.3 to represent the edible portion of the pig. This allowed for estimation of the proportion of shared pork that was eaten at home versus shared for each village.

**Parameter**	**Estimate**	**LL**	**UL**
Total sacrificed pigs whose meat was SHARED/SOLD	221		
**Number of kg of pork (pigs*50kg*0.3)**	7735kg		
**Number of kg of pork purchased from WITHIN**	1545.5kg		
**Proportion of pork eaten by HOUSEHOLD**	80% (6189.5 / 7735kg)	0%	83.9%

Travel-related parameters: A section of the survey asked respondents to list all trips taken in the past 4 months by any member of the household for which the traveler spent at least 1 night outside of the village. The person, location, and duration of each trip were recorded. In analysis, destinations were evaluated to determine if they were endemic for *T. solium* transmission. Given that travel was likely to be underreported and most destinations were endemic areas, we chose not to exclude non-endemic areas. Final parameter values were determined by averaging the results of the two survey time-points. The "plausible range" was determined by selecting the minimum and maximum values from among the seven villages surveyed.

traveler-prop: The proportion of households that have a traveler was determined by dividing the number households that reported at least one trip by the total number of households.

**Parameter**	**Estimate**	**LL**	**UL**
Number of households	828		
**At least 1 TRIP ("TRAVELER HH")**	42.3% (350/828)	24.6%	65.4%

travel-freq: The frequency of travel among travelers was determined by counting the total number of trips completed by each household in the past 4-months, and using this to determine the mean number of trips per week and the mean interval between trips.

**Parameter**	**Estimate**	**LL**	**UL**
Number of travelers	350		
MEAN # of trips in 4-month period	2.16	1.08	3.5
MEAN # of trips / week	0.12	0.06	0.20
**MEAN frequency of trips (weeks between trips)**	8.00	16.02	4.94

travel-duration: The mean duration of travel was determined by averaging the duration of every trip reported by a traveling household. Final travel-durations were analyzed to determine the probability distribution and summary statistics to best describe them. An exponential distribution was chosen as the optimal fit by comparing AIC values for evaluated distributions.

**Parameter**	**Estimate**	**LL**	**UL**
Number of travelers	350		
MEAN trip duration (days)	12.28	5.9	23.5
**MEAN trip duration in weeks:**	1.75	0.84	3.36

SOURCE #3: GPS pig tracking study (GPS)

Model parameters:

latrine-use	Among households with access to latrines, proportion that are in "good" condition and are "always" used by all household inhabitants
cont-radius	Distance from household at which open defecation occurs among household not using latrines
corral-always	Among households that raise pigs and own pig-corrals, the proportion of corrals that are in "good" condition and are "always" used to contain all pigs
corral-sometimes	Among households that raise pigs and own pig-corrals, the proportion of corrals that are "sometimes" used to contain pigs

prop-corral-some	Among pigs raised in households that "sometimes" contain pigs in corrals, the proportion of pigs contained at any given time
home-range	Radius of the area pigs cover when roaming (i.e., not contained in corrals), and within which exposure to *T. solium* is assumed to occur

Description of study:
A detailed description of this study is can be found in Chapter 3. The study consisted of two separate activities, both carried out in 2018 in three rural villages of northern Peru (Piura region). First, we conducted a door-to-door survey of all households in the villages. The survey asked adult heads-of-household about the presence and use of latrines and pig-raising practices. Second, we conducted GPS tracking of a sample of free-roaming pigs. Overall, we tracked 108 pigs for 6 days each, and included GPS tracking in both the rainy and dry season. Roaming patterns were then analyzed to determine the size of each pig's "home-range."

Methods/results:
<u>*latrine-use:*</u> Heads-of-households were asked if their household had access to a latrine/indoor bathroom, or if they used outdoor areas to defecate. If a latrine was present, we then asked how often members of the household used the latrine, giving the options "always," "sometimes," or "never." The option of "always" was only recorded if *all* members of the household, including children, were reported to *always* use the latrine/bathroom. We also inspected the condition of the latrine, and recorded it as "good," "normal," or "bad," and inspected the areas around the house for evidence of feces or soiled paper. In the model, "latrine-use" was defined as the proportion of households with latrines that reported "always" using latrines, and for which latrine condition was "good" and no evidence of feces was observed in the household area. The final parameter value was determined by averaging the results of the three villages. The "plausible range" was determined by selecting the minimum and maximum values from among the villages surveyed.

Among households with latrines, the proportion of HH's that are in GOOD condition and are used ALWAYS		
**Estimate**	**LL**	**UL**
73% (142 / 194)	57%	86%

<u>*cont-radius*:</u> For households that reported the practice of open defecation, respondents were asked to indicate the location of the defecation area, and a GPS point was recorded at the location where feces or soiled paper was visualized. We then calculated the distance between the open defecation point and the household (GPS point taken at the front door), and labeled this distance as the "contamination radius" for each house. Final contamination radii were analyzed to determine the probability distribution and summary statistics to best describe them. A log-normal distribution was chosen as the optimal fit by comparing AIC values for evaluated distributions. The "plausible range" was derived from a 95% confidence interval of the log-normal distribution. When applied in the model, tapeworm carriers will shed infectious *T. solium* eggs and proglottids onto a location that is determined by generated a random value from the *cont-radius* distribution and applying a random angle from the household location.

	**Estimate (m)**	**LL**	**UL**
Log-mean	3.27	3.14	3.40
**exp(log-mean)**	**26.3**	**23.0**	**30.1**
**Log-SD**	**0.546**		

<u>*corral-always, corral-sometimes:*</u> For households that reported raising pigs, pig-owners were asked if they allowed their pigs to roam freely or kept their pigs contained in corrals. A response of "always" indicated that *all* pigs (including piglets) were *always* kept enclosed (or tied). Reports were confirmed or

adjusted based on observation of pig-owners current practices, and were further validated by cross-referencing reports with pig-level data collected in the same month in a separate pig sero-survey (each pig reported as "free" of "enclosed"). The "plausible range" was determined by selecting the minimum and maximum values from among the villages surveyed. For application in the model, pig-raising households that possessed corrals were assigned as either "always," "sometimes," or "never" users of corrals. Pigs in households that "sometimes" used corrals were either corralled or not corralled depending on *prop-corral-some*.

	**Estimate**	**LL**	**UL**
**Always**	35% (36 / 102)	33%	39%
**Sometimes**	57 % (58 / 102)	53%	62%
**Never**	8% (8 / 102)	4%	13%

prop-corral-some: The proportion of pigs that were corralled among households that "sometimes" corral their pigs was determined by cross-referencing survey responses (household-level) with pig-level data from a serological survey that took place in the same month. Households that reported "sometimes" using corrals were found in the serological data, and the numbers of pigs that were "contained" vs. "free-roaming" at the time of the sero-survey were recorded. The "plausible range" was determined by selecting the minimum and maximum values from among the villages surveyed.

	**Estimate**	**LL**	**UL**
Total pigs in "sometimes" households	261		
**Contained**	32% (83 / 261 pigs)	15%	44%
**Free-roaming**	68% (178 / 261 pigs)	56%	85%

home-range: The area of each pig's "home-range" was calculated using the Localized Convex Hulls (LoCoH) home range algorithm. Home-range areas represent the area of active foraging that represents the densest 90% of a pigs range. The total area (m^2) was calculated for each pig, and was converted to a radius that assumed each range followed a circular shape. Final home-range radii were analyzed to determine the probability distribution and summary statistics to best describe them. A log-normal distribution was chosen as the optimal fit by comparing AIC values for evaluated distributions. In the sub-analysis, a significant difference between villages and seasons was detected. Therefore, upper and lower limits were determined by extracting the mean-log from the largest and smallest village/season combinations.

	**Estimate**	**LL**	**UL**
**mean-log**	**3.79**	**3.39**	**4.56**
exp(mean-log)	44.2	29.7	95.5
**sd-log**	**0.552**		

SOURCE #4: Literature review and expert opinion

Model parameters:

import-prev	Proportion of imported pigs that have cysticercosis
light-to-heavy	Proportion of infected imported pigs that have light vs. heavy cyst infection
travel-incidence	Rate of human taeniasis infection during travel to external endemic areas
tn-incubation	Latency period after initial infection (taeniasis) before beginning to expel eggs
tn-lifespan	Mean duration of taeniasis infections
decay-mean	Mean time eggs remain viable in the environment before decaying

import-prev: The proportion of imported pigs that had cyst infection was assumed to be consistent with the prevalence of cyst infection expected in a standard endemic village of northern Peru. We therefore estimated this prevalence using a necropsy study conducted in this region of Peru in 2017 [75]. Since only a sample of seropositive pigs were necropsied in this study, but all pigs were tested with the EITB assay, we estimated the total number of infected pigs by applying the proportion infected at each EITB band level (1-7 positive bands) to the total number of pigs at each band level. Given the limited data for this parameter, the "plausible range" of 0 to 30% was set manually based on plausible limits determined by our expertise.

WB # bands	Prevalence of cyst infection in necropsy sample	Pig population at band level (n=828 pigs)	Expected # with cyst infection
0	0/0 = 0%	395/828 = 47%	395*0 = 0
1	2/22 = 9.1%	87/828 = 10.5%	87*0.091= 7
2	4/42 = 9.5%	127/828 = 15.3%	127*0.095 = 12
3	17/52 = 32.7%	142/828 = 17.2%	142*.327 = 46
4	6/18 = 33.3%	37/828 = 4.5%	37*0.333 = 12
5	6/10 = 60%	16/828 = 1.9%	16*0.60 = 10
6	5/5 = 100%	9/828 = 1.1%	9*1.0 = 9
7	9/9 = 100%	15/828 = 1.8%	15*1.0 = 15
**TOTAL**	**49 / 158 = 31%**	**433 / 828 pigs = 52%**	**111 / 828 = 13.4%**

light-to-heavy: The proportion of infected imported pigs that had light vs. heavy cyst infection was based on the same necropsy study referenced above [75]. Among infected pigs that were found on necropsy, those with < 100 viable cysts were considered lightly infection and those with ≥ 100 viable cysts were heavily infected. Given the limited data for this parameter, the "plausible range" of 50% to 100% of infected imported pigs with *light* infection was set manually based on what our experts believed could be the plausible limits.

Parameter	Estimate
Total pigs necropsied	158
Necropsy-positive (1+ cyst)	49
**Light infection (1-99 cysts)**	**75.5% (37 / 49)**
**Heavy infection (>100 cysts)**	**24.5% (12 / 49)**

travel-incidence: The incidence of human tapeworm infections (taeniasis) among travelers traveling to other endemic areas was assumed to be the same as the incidence observed in a standard endemic village. However, the incidence of taeniasis in endemic villages is unknown and has not been published. We therefore estimated taeniasis incidence based on the known prevalence in this region and an estimate of the duration of infection using the equation: Incidence = Prevalence / Duration. The prevalence was based on a cross-sectional study of tenaisis prevalence conducted in this region [75]. See below for explanation of taeniasis duration used for this calculation (2 years). The "plausible range" was determined by calculating incidence with the minimum and maximum prevalences observed among the 7 villages included in the original study. Taeniasis incidence was then applied in the model as the probability of infection per person per week of travel.

Parameter	Estimate	LL	UL
Taeniasis prevalence	2.4% (34/1420)	0.9% (2/218)	5.3% (8/151)
Taeniasis duration	104 weeks	26 weeks	208 weeks
**INCIDENCE (probability of infection per week)**	**0.024 / 104 = 0.000231**	**0.009 / 208= 0.000043**	**0.053 / 26 = 0.0020**

tn-incubation: The period of time after initial ingestion of a viable *T. solium* cyst and growth of a mature tapeworm that expels infection eggs and proglottids is widely reported in literature to be approximately 2 months (8 weeks). See [22,26,27]. No "plausible range" or probability distribution was utilized for this fixed parameter value.

tn-lifespan: Humans that are infected with tapeworm will have a natural duration of infection that is determined by this parameter. Taeniasis duration is unknown and has not been studied due to ethical barriers. A range of 2-4 years is typically reported, and is based on age-specific prevalence data, biological plausibility, and auto-infection of a scientist in 1935. See [Garcia, Yoshino]. A zero-truncated normal distribution with standard deviation of 50 weeks was chosen for simplicity, but is not supported by data. In the model, the duration of each individual tapeworm infection is randomly drawn from this distribution.

**Estimate**	**LL**	**UL**
Mean = 104 weeks (2 years)	26 weeks (0.5 years); Truncated at 0	208 weeks (4 years)
SD = 50	SD = 50<br>95% LL = 0<br>95% UL = 132 weeks (2.5 years)	SD = 50<br>95% LL = 125 weeks (2.4 years)<br>95% UL = 290 weeks (5.6 years)

decay-mean: If a tapeworm carrier is practicing open defecation, they will contamination their environment with *T. solium* eggs and proglottids. After the infection ends, eggs (but not proglottids) will persist in the environment until they naturally decay. The longevity of eggs in the environment is determined by this parameter. Estimates for the longevity of eggs in the environment are based on experimentation done on eggs of related species in the 1970s [134]. These experiments indicate that longevity is impacted by temperature and moisture. The mean and plausible range was derived from observations from a variety of experiments conducted in different conditions. In the model, a fixed probability of decay is applied each week, which creates an exponential survival function with the mean inversely related to the probability of decay.

**Eggs decay in the environment according to the EXPONENTIAL SURVIVAL FUNCTION, where at each time (t) there is a constant probability of decay (λ), the survival function is therefore given by f(t) = exp(-λt), with mean decay time given by E(t) = 1/λ**		
**Estimate:**	**LL**	**UL**
**λ = 0.125**	**1.0**	**0.038**
E(t) = 8 weeks	1 week	26 weeks
