# Supplementary material for: Understanding transmission and control of the pork tapeworm with CystiAgent: a spatially explicit agent-based model
Source: Parasit Vectors. 2020 Jul 24;13:372. doi: 10.1186/s13071-020-04226-8 (PMC7379812; doi:10.1186/s13071-020-04226-8)
Supplement: Supplementary file 2 — Additional file 2: Text S2. Supplemental methods and results for Sobol’ variance decomposition. Figure S1. Graphical results of Sobol’ variance decomposition for crude and calibrated model versions on the medium-density village. [file 13071_2020_4226_MOESM2_ESM.pdf]

Additional file 2. Text S2. Supplemental methods and results for Sobol' variance decomposition.

Methods

Readers seeking a detailed description of the Sobol' methodology should look to seminal works by Sobol' [1,2] and in-depth examples of their application to complex models [3–5]. For our application of the Sobol' method, we first determined plausible ranges for each model parameter as described above, and sampled values from each parameter distribution using a Sobol' sequence. Compared to other common sampling methods (e.g., simple random, Latin-hypercube, etc), a Sobol' quasi-random sequence has been found to cover the parameter space more efficiently and allows for smaller sample sizes in sensitivity analyses [6,7]. As described in [8], the sample was divided into two input matrices, and then further arranged into $k + 2$ design matrices for evaluation in the model. The computational cost of this method depends on the number of input parameters (k) and the chosen number of samples drawn for each parameter (N), totaling $N*(k + 2)$. For this analysis, we selected per-parameter sample sizes (N) of 5000, 1500, and 1500 for the low, medium, and high-density villages, respectively, to account for the extra computing time required for larger populations. $N \geq 500$ is recommended for complex models [9]. Evaluating $k = 33$ model parameters in the full model and $k = 22$ parameters in the reduced model led to final computational costs between 36,000 and 175,000 per analysis, depending on the village.

The results of model simulations were analyzed using the "sobol2007" function available in the "sensitivity" package in R. The Sobol' method quantifies sensitivity of the model to each parameter with two measures: first-order sensitivity index (S_i), and a total effects sensitivity index (ST_i). S_i estimates the independent contribution of each parameter to variance in the model outcomes, while ST_i estimates the full contribution of each parameter after considering interactions with other parameters [6,10]. Equations for S_i and ST_i are given below, with V_t representing the overall variance in the output, V_i representing the variance due to the uncertainty in parameter i, and $S_{(-i)}$ representing the sum of all S_i indices other than index i. First-order indices were considered significant if $S_i > 0.02$ in the full model analysis, or $S_i > 0.01$ in the reduced model analysis; 95% confidence intervals for S_i and ST_i were generated with 100

bootstrapped replications [11]. First-order and total-effect indices were calculated for human taeniasis and porcine cysticercosis in each of the three villages analyzed.

$$S_i = V_i / V_t$$

$$ST_i = 1 - S_{(-i)}$$

Results

Figure S1 contains graphs of CystiAgent model parameters that had significant first-order (S_i) and total-effect (ST_i) indices in Sobol' sensitivity analyses.

Full model analysis. Of the 33 parameters included in the analysis of the full CystiAgent model, parameters that were consistently identified as impactful on rates of porcine cysticercosis were the parameters defining the use of corrals to contain pigs, and pig-related tuning parameters. Specifically, "always" using corrals for all owned pigs ("corral-always") had the most consistently high impact on output variance, with first-order indices of S = 0.10, 0.35, and 0.27 in low, medium, and high-density villages, meaning that 10%, 35%, and 27% of the variance in pig infection was attributed to the uncertainty range of this parameter. Similarly, the probability of light-infection after exposure to *T. solium* eggs ("light-inf") was highly impactful in each of the village analyses, with first-order indices of 0.25, 0.44, and 0.27 in the three villages.

The parameters that contributed most to variance in rates of human taeniasis in the full model analysis were those that determined the number of pigs in the population (and therefore more opportunities for infection) and the set of human-related tuning parameters. Specifically, the proportion of households raising pigs ("prop-pig-owners"), the mean number of pigs per household ("pigs-per-hh"), the proportion of pigs sold prior to slaughter ("pigs-sold"), and the proportion of sold pigs that were exported out of the village ("pigs-export") all had significant first-order indices in at least two of the three villages tested. For human-related tuning parameters, the probabilities of tapeworm infection after slaughter of a lightly ("pl2h") or heavily ("ph2h") infected pig were both highly impactful. The mean duration of

tapeworm infections was also an important contributor to output variance in in two of the three villages ("tn-lifespan").

Reduced model analysis. When tuning parameters and village input characteristics were fixed for the reduced model analysis, the relationships between the remaining model parameters and model outputs changed considerably. Of the 22 parameters included in the reduced model analysis, the most consistently impactful parameter for both porcine cysticercosis and human taeniasis was the average duration of taeniasis infection ("tn-lifespan"), which accounted for 31%, 39%, and 29% of the total variation in pig infections, and 18%, 16%, and 17% of the total variation in human taeniasis rates across the three villages tested. After tapeworm lifespan, the second and third most impactful parameters in the reduced model analysis were the size of pig home-ranges ("home-range") and use of latrines ("latrine-use"), neither of which were identified as impactful in the full model analysis. These parameters accounted for an average of 11% and 8% of the variance in pig infection, and 5% and 4% of variance in human taeniasis, respectively, across the three villages evaluated. Finally, the proportion of pigs exported ("pigs-exported") and sold ("pigs-sold") were consistently identified as impactful parameters in the reduced model analysis.

Total effect indices. Total-effect indices (ST_i) in the full model analysis followed similar patterns as first-order effects (S_i), but were consistently larger to account for the extra variance due to interactions between parameters. Of the three test villages, the low-density village had the greatest disparity between first-order and total-effect indices. Similarly, total effect indices were greater in the reduced model analysis compared to the full model analysis, indicating that interaction effects between parameters contributed to a greater proportion of output variance in the reduced model.

References

1. Sobol M. Sensitivity estimates for nonlinear mathematical models. Math Model Comput Exp. 1993;1(4):407–14.
2. Sobol IM. Global sensitivity indices for nonlinear mathematical models and their Monte Carlo estimates. Math Comput Simul. 2001;55:271–80.
3. Ligmann-Zielinska A, Kramer DB, Cheruvelil KS, Soranno PA. Using uncertainty and sensitivity analyses in socioecological agent-based models to improve their analytical performance and policy relevance. PLoS One. 2014;9(10). PMID: 25340764
4. Zhang XY, Trame MN, Lesko LJ, Schmidt S. Sobol sensitivity analysis: A tool to guide the development and evaluation of systems pharmacology models. CPT Pharmacometrics Syst Pharmacol. 2015;4(2):69–79. PMID: 27548289
5. Fonoberova M, Fonoberov VA, Mezić I. Global sensitivity/uncertainty analysis for agent-based models. Reliab Eng Syst Saf. 2013;118:8–17.
6. Homma T, Saltelli A. Use of Sobol's quasirandom sequence generator for integration of modified uncertainty importance measure. J Nucl Sci Technol. 1995;32(11):1164–73.
7. Kucherenko S, Albrecht D, Saltelli A. Exploring multi-dimensional spaces: a comparison of Latin Hypercube and Quasi Monte Carlo sampling techniques. Cornell University Library. 2015 (available from https://arxiv.org/abs/1505.02350). p. 1–30.
8. Saltelli A, Annoni P, Azzini I, Campolongo F, Ratto M, Tarantola S. Variance based sensitivity analysis of model output. Design and estimator for the total sensitivity index. Comput Phys Commun. 2010;181(2):259–70. PMID: 15246654
9. Convertino M, Muñoz-Carpena R, Chu-Agor ML, Kiker GA, Linkov I. Untangling drivers of species distributions: Global sensitivity and uncertainty analyses of MaxEnt. Environ Model Softw. 2014;51:296–309.
10. Chan K, Saltelli A, Tarantola S. Sensitivity analysis of model output: variance-based methods make the difference. In: Proceedings of the 1997 Winter Simulation Conference. 1997. p. 261–8.
11. Iooss B, Janon A, Pujol G. Package "sensitivity": Global sensitivity analysis of model outputs. 2018 (available from https://cran.r-project.org/web/packages/sensitivity/sensitivity.pdf).

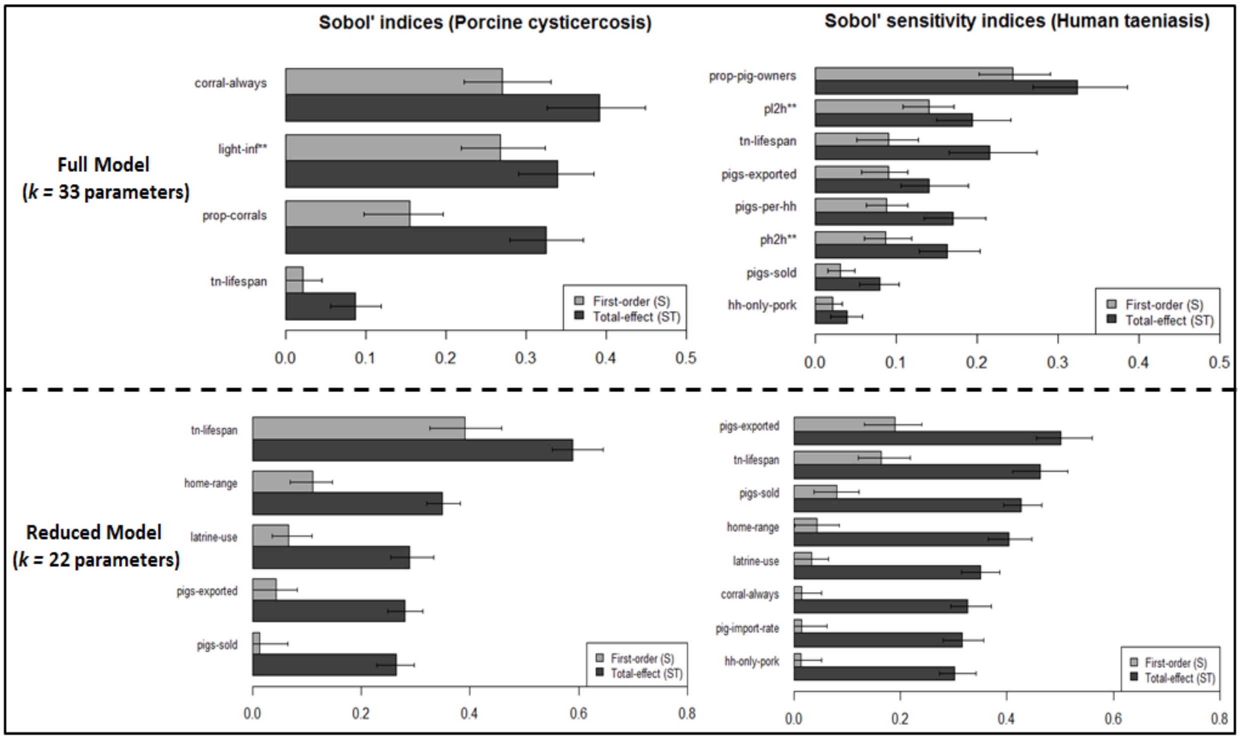

Fig S1. Sobol' first- and total-order indices for porcine cysticercosis (left) and human taeniasis (right), in the full model (top) and reduced model (bottom), medium-density village. Parameters with first-order indices $S_i > 0.02$ in the full model analysis and $S_i > 0.01$ in the reduced model analysis are shown. 95% confidence intervals produced with 100 bootstrap replications. See Table 1 (main text) for descriptions of parameter names and functions.
