# Supplementary material for: Understanding transmission and control of the pork tapeworm with CystiAgent: a spatially explicit agent-based model
Source: Parasit Vectors. 2020 Jul 24;13:372. doi: 10.1186/s13071-020-04226-8 (PMC7379812; doi:10.1186/s13071-020-04226-8)
Supplement: Supplementary file 3 — Additional file 3: Figure S2. Latin hypercube sampling-partial rank correlation coefficient (LHS-PRCC) results of crude and calibrated models across low, medium, and high-density villages. Parameters with significant LHS-PRCC coefficients (P < 0.0015) are shown. [file 13071_2020_4226_MOESM3_ESM.pdf]

Additional file 3: Figure S2. Latin hypercube sampling-partial rank correlation coefficient (LHS-PRCC) results of full and reduced models across low, medium, and high-density villages. Parameters with significant LHS-PRCC coefficients (p< 0.0015) shown. See table 1 (main text) for description of model parameters).

	Full Model (k = 33 parameters)						Reduced Model (k = 22 parameters)					
**Parameters**	High		Medium		Low		High		Medium		Low	
**Pig**												
tuning-pig§	●●●	■■	●●●	■■	●●●	■■■						
corral-always	●●●	■■	●●●	■■	●●●	■■	●●	■■	●●	■	●	+
prop-corrals	●●●	■	●●●	■■	●●●	■						
corral-sometimes	+		●	+	●	+	●	+	+	+	+	
prop-corral-some	+		●●	+	●	+	●	+	+	+	+	+
home-range	+	+	+	+	●	+	●●●	■■■	●●●	■■	●●	■■
latrine-use	+		+	+	●	+	●●	■■	●●	■■	●●	■■
humans-per-hh	+	+	+	+	●	+						
prop-latrines	+		+	+	+	+						
slaughter-age		+		+		+	+		+	+		+
decay-mean					+		+	+	+	+	+	+
cont-radius							+		+	+		
home-range-sd							+	+	+	+	+	+
**Human**												
tuning-human§	■■■	+	■■■	●	■■■	●●						
prop-pig-owners	■■■	+	■■■	+	■■■	●●						
tn-lifespan	■■■	+	■■■	●	■■	●●	■■■	●●●	■■■	●●●	■■	●●●
pigs-per-hh	■■■	+	■■■	+	■■	●						
pigs-exported	■■■	+	■■■	+	■■	●	■■■	●●	■■■	●●	■■	●●
pigs-sold	■■	+	■■	+	■■	●	■■	●●	■■	●●	■■	●●
hh-only-pork	■		■		■	+	+	+	■	+	+	+
shared-pork-hh	■		■		■	+	■	+	■	+	+	+
pig-import-rate	■		+	+	+	+	■■	●	■	●	■	●
import-prev	■		■		+	+	■■	●	■■	●	■	●
sold-pork	+		+		+		+	+	+	+		
travel-duration	+											
travel-incidence							+	+			+	+
light-to-heavy							+		+	+	+	+
traveler-prop									+	+		

■ = Human taeniasis, ● = Porcine cysticercosis
SOBOL first-order indices (S_i) : ■■■ / ●●● > 0.25; ■■ / ●● > 0.1; ■ / ● > 0.02
PRCC (absolute-value of ρ) : ■■■ / ●●● > 0.5; ■■ / ●● > 0.25; ■ / ● > 0.1; + / + < 0.1; (all p < 0.0015)
§tuning-pig (4 parameters) and tuning-human (2 parameters) represent the combined impact of each set.
